# AI snake oil? A risk/benefit analysis for toxicology

**DOI:** 10.3389/frai.2026.1779276

**Published:** 2026-05-19

**Authors:** Thomas Hartung, Mohan Rao, Mamta Behl, Alexandra Maertens

**Affiliations:** 1 Doerenkamp-Zbinden Chair for Evidence-Based Toxicology, Center for Alternatives to Animal Testing (CAAT), Johns Hopkins University, Baltimore, MD, United States; 2 CAAT-Europe, University of Konstanz, Konstanz, Germany; 3 CAATevents gGmbH, Solingen, Germany; 4 Sanofi, Preclinical Safety, Translational Medicine Unit, Cambridge, MA, United States; 5 Neurocrine Biosciences Inc., San Diego, CA, United States

**Keywords:** artificial intelligence, Green Toxicology, new approach methodologies, predictive toxicology, regulatory science, risk assessment, toxicology, validation

## Abstract

Artificial intelligence (AI) is increasingly used to support predictive, mechanistic, and human-relevant toxicology at scale. However, its integration into regulatory science - particularly in drug development - remains uneven, because encouraging technical performance has not yet translated automatically into regulatory trust. Representative AI toxicology studies now span datasets from roughly 10^3^ chemicals to >3 × 10^4^ peptide or chemical records and report performance ranging from modest in prospective screening settings to strong on narrower, well-curated endpoints. This manuscript presents a critical analysis of the dual nature of AI in toxicology. We review the state of the art in AI-enabled applications, ranging from Green Toxicology and the Human Exposome to specific challenges in safety assessment for biologics and synthetic peptides. Particular attention is given to the gap between rapid model development and regulatory acceptance, highlighted by the challenges of model interpretability, dataset bias, insufficient external validation, and the assessment of complex endpoints like immunogenicity. To navigate these complexities, we discuss the next-generation “*e-validation*” framework and emphasize the TREAT principle - Trustworthiness, Reproducibility, Explainability, Applicability, and Transparency - as a foundation for building regulatory trust. We hypothesize that AI-based methods in toxicology can achieve regulatory acceptance when they satisfy the TREAT criteria and undergo continuous e-validation within a clearly defined context of use. This framework distinguishes credible AI applications from “*snake oil*” by establishing measurable criteria for trust-building, including dataset provenance, external validation, uncertainty characterization, and life-cycle monitoring. We argue that AI is neither a miracle cure nor a technological illusion, but a powerful evidence engine that can contribute to a more predictive and ethical toxicological science when it is rigorously validated.

## 1 Introduction

The term “*snake oil*”^1^ refers to a product - typically a medicine or remedy - that is promoted as having remarkable or miraculous effects but is actually worthless or fraudulent. In modern language, it’s a metaphor used for deceptive marketing, scams, or health care fraud where someone promises solutions that do not work or have no scientific backing. The term can also be used figuratively for any item or solution that is sold with extravagant claims but lacks real value or effectiveness.

Historically, the phrase comes from the 19TH-century United States, where so-called “*snake oil salesmen*” traveled around selling supposed cure-all elixirs. These products were often marketed as remedies for a wide range of ailments, but usually lacked either genuine snake oil or other effective medicinal ingredients. Over time, this association made “*snake oil*” synonymous with fake remedies and fraudulent health products more generally.

The phrase “*snake oil*” evokes quackery: extravagant claims with little proof. In current AI discourse, especially in life sciences and toxicology, similarly inflated narratives sometimes appear when model performance is discussed without context of use, dataset quality, or validation. The real question is therefore not whether AI is impressive in principle, but what chain of evidence is needed before its outputs can support credible scientific or regulatory decisions.

**Figure 1:**
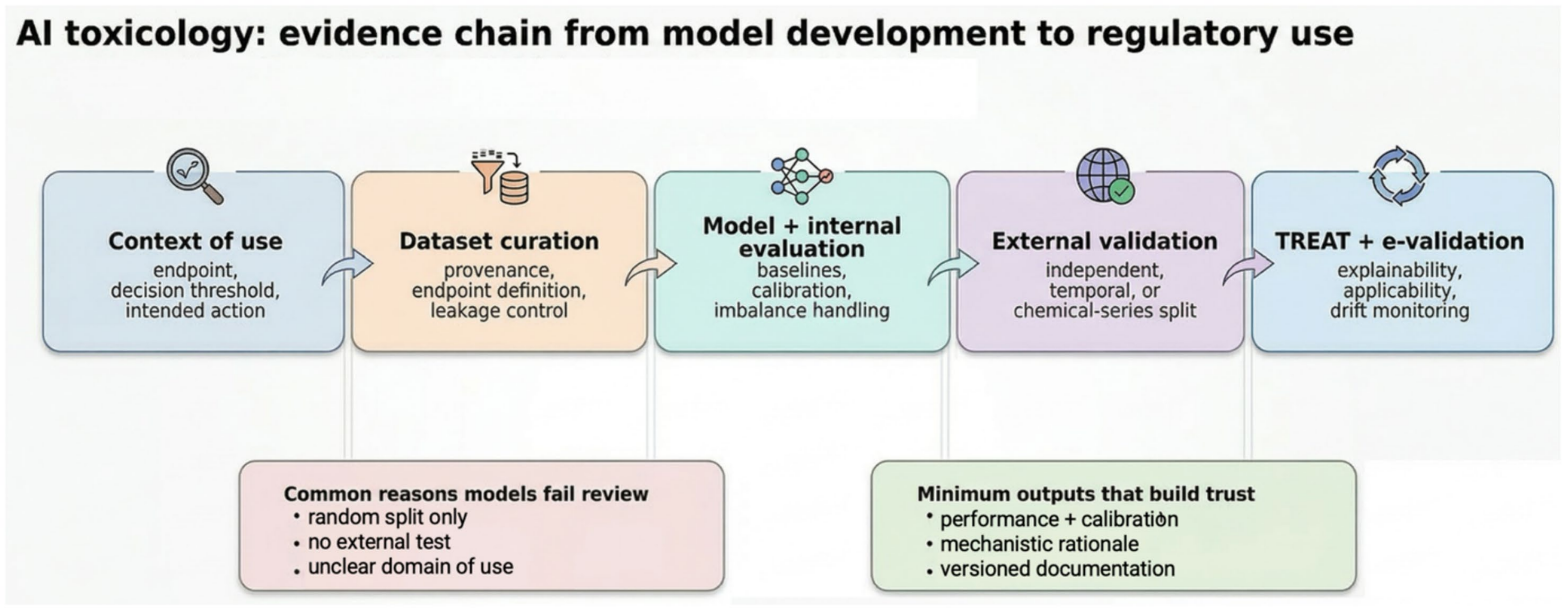
Evidence chain from AI model development to regulatory use in toxicology. A research-oriented workflow emphasizing context of use, dataset curation, internal and external validation, and life-cycle monitoring under TREAT and e-validation. Figure draft created with Gemini Nano Banana 2.

But this comparison is not entirely fair. In toxicology, AI already offers meaningful value when developed and deployed responsibly. Figure 1 summarizes the evidence chain that separates promising AI from over claim: a defined context of use, auditable datasets, appropriate internal and external validation, and life-cycle monitoring under TREAT and e-validation. The purpose of this commentary is therefore to examine the dual nature of AI in toxicology - as both a potential disruptor of outdated practices and, if misapplied, a vessel of technological overpromise.

### 1.1 Perspective scope and literature basis

This article remains a Hypothesis and Theory/Perspective paper rather than a formal systematic review. To make the evidentiary basis more transparent, we conducted structured scoping searches in PubMed, Scopus, and Web of Science between January 2025 and February 2026 using combinations of the terms “*artificial intelligence*,” “*machine learning*,” “*deep learning*,” with “*toxicology*,” “*risk assessment*,” “*new approach methodologies*,” “*validation*,” “*immunogenicity*,” and “*virtual control group*.”

We prioritized English-language publications that (i) addressed toxicological endpoints or regulatory decision support, (ii) reported a defined dataset, use case, or framework, and (iii) described evaluation metrics, external validation, mechanistic interpretation, or governance considerations when available. This strategy was intended to anchor the perspective in representative evidence, not to deliver a PRISMA-style exhaustive synthesis.

## 2 The opportunity: redefining toxicology through AI

**Figure 2:**
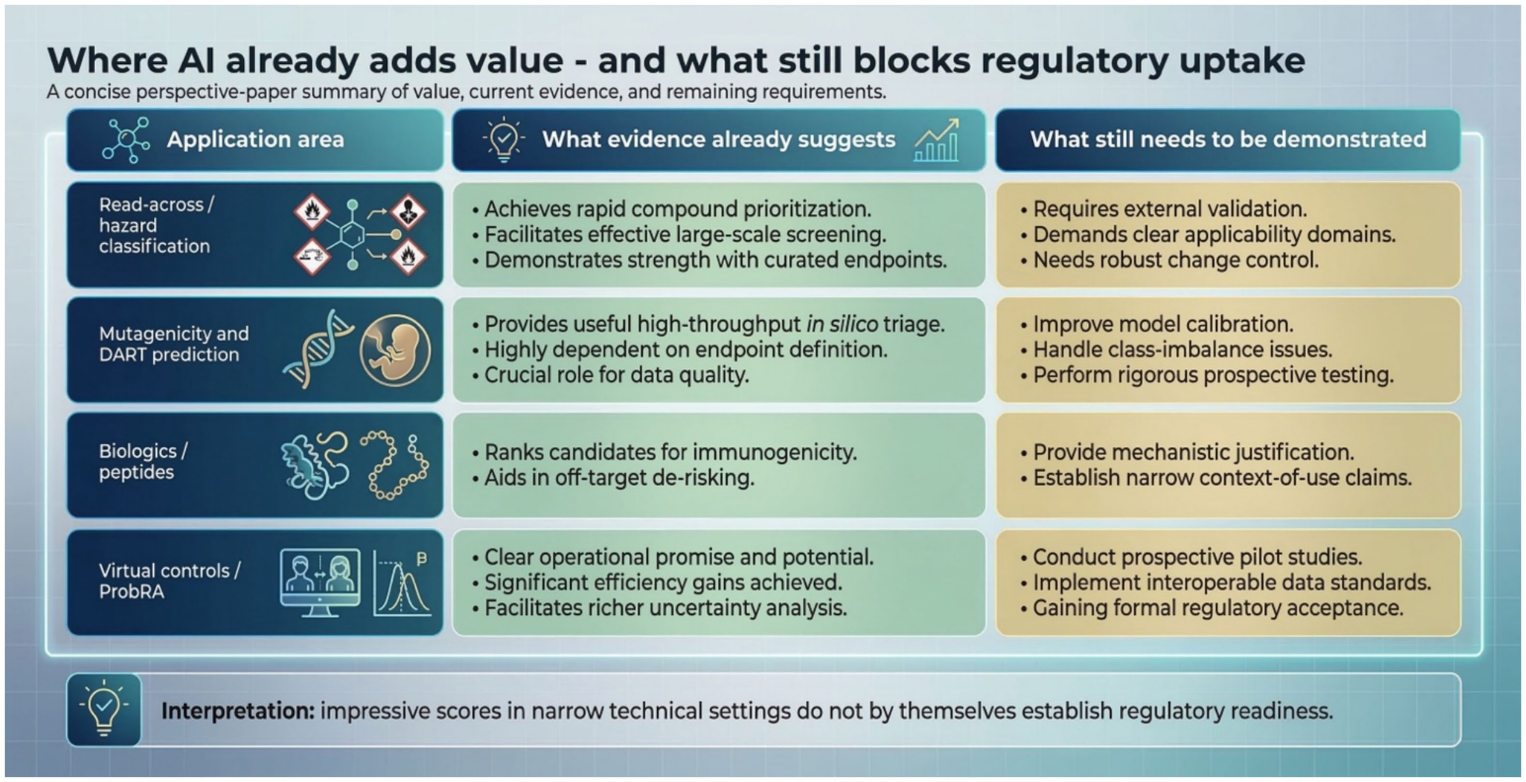
Where AI already adds value - and what still blocks regulatory uptake. A concise perspective-paper summary of application areas in which evidence is accumulating and the additional controls needed before routine regulatory use. Figure draft created with Gemini Nano Banana 2.

Toxicology is undergoing a rapid methodological shift, driven by new approach methodologies (NAMs), expanding biological and chemical data streams, and increasing demand for animal-free, human-relevant models (Schmeisser et al., 2023; Tsaioun et al., 2025; Luechtefeld and Hartung, 2025). Recent broad reviews, including Alam et al. (2025), catalog the growing AI and generative-AI literature in toxicology. Here, however, the focus is narrower: what would make such systems sufficiently trustworthy for real regulatory or public-health decisions? Figure 2 summarizes where the evidence is strongest and where key barriers remain.

AI-driven models are already delivering concrete benefits (Hartung, 2023b, 2023d). Automated read-across frameworks are being used to predict chemical toxicity based on structural and biological similarity (Luechtefeld and Hartung, 2025). Deep learning systems have demonstrated competitive or improved predictive performance relative to traditional approaches for specific endpoints such as mutagenicity and reproductive toxicity (Gini et al., 2019; Lee et al., 2025). Moreover, probabilistic risk assessment (ProbRA) has emerged as a powerful application area for AI (Maertens et al., 2022, 2024a, 2025; Jang et al., 2022). Rather than producing binary yes/no decisions, AI models can generate risk distributions and uncertainty estimates, enabling more nuanced regulatory decisions.

A most promising application is the creation of virtual control groups by AI with the potential to save 15–20% of animals (Steger-Hartmann et al., 2020; Golden et al., 2024). The VICT3R project^2^ of the Innovative Health Initiative is currently exploring this option.

In evidence-based toxicology (EBT),^3^ AI accelerates systematic reviews by extracting, standardizing, and synthesizing evidence across thousands of publications (Hartung and Tsaioun, 2024). Tools like SysRev^4^ (Bozada et al., 2021) and natural language processing (NLP)-based pipelines now enable partial automation of risk-of-bias assessments (Hartung et al., 2025b), literature mining, and meta-analyses. The ongoing explosive growth in AI capabilities promises further advances relevant to toxicology (Luechtefeld and Hartung, 2025). AI also facilitates mechanistic modeling through integration with frameworks like adverse outcome pathways (AOPs) (Leist et al., 2017), creating causal maps that link molecular perturbations to phenotypic outcomes. In the ONTOX^5^ project and others like RISK-HUNT3R^6^ and PANORAMIX,^7^ AI enables synthesis of literature, ~omics, and *in vitro* assay data to develop non-animal approaches for systemic toxicity assessment.

### 2.1 AI in drug development: opportunities and challenges

The U. S. Food and Drug Administration (FDA), through the FDA Modernization Act 2.0 and its Roadmap to Reducing Animal Testing in Preclinical Safety Studies, has begun creating a clearer pathway for incorporating non-animal evidence into preclinical safety assessment (FDA, 2022, 2025a, 2026). The NIH has similarly launched the Complement Animal Research In Experimentation (Complement-ARIE) program^8^ to develop, standardize, validate, and use NAMs that more accurately model human biology. Similar developments are underway in Europe and the UK, including the recent UK roadmap and ongoing EU policy work (Hansell et al., 2024; Hansell and Hartung, 2025a, 2025b; Hartung, 2026).

Despite this progress, a significant gap remains between the rapid development of AI-based NAMs and their regulatory acceptance. Many models are generated in academic or industrial silos using datasets that lack standardization, biological relevance, or rigorous quality control. Even the FDA’s rollout of Elsa, an internal large language model assistant designed to help staff read, write, and summarize regulatory materials, illustrates both the promise of AI in regulatory operations and the need for careful governance, performance monitoring, and human oversight (FDA, 2025b). Without rigorous validation, even high-tech tools risk being perceived as digital “*snake oil*” - impressive in function but opaque in reliability.

Without alignment to regulatory expectations and interpretability frameworks, even technically advanced models may be considered unreliable. Clearer FDA guidance on validation criteria and performance requirements will be critical to establishing the credibility of AI-enabled NAMs and facilitating their broader integration into regulatory decision-making.

To be considered credible and useful in regulatory contexts, AI-generated results, such as predictions about toxicity or organ-specific effects, must be presented in formats that regulators recognize and trust. This includes using standardized templates and ensuring that predictions are based on well-understood biological mechanisms. However, many AI models are optimized for performance metrics rather than regulatory compatibility, which limits their practical utility. Incorporating mechanistic insights that align with adverse outcome pathways (AOPs) can improve biological relevance, but this alone may not be sufficient. The TREAT framework - Trustworthiness, Reproducibility, Explainability, Applicability, and Transparency - has emerged as a guiding principle for regulatory AI (Hartung et al., 2025a) and is conceptually aligned with the recent FDA/EMA good AI practice principles for drug development (FDA and EMA, 2026), though applying these standards consistently across different AI platforms remains a work in progress.

Toxicological evaluation is shaped by the type of therapeutic modality, with small molecules and biologics governed primarily by ICH M3(R2) and ICH S6(R1) (ICH, 2011), respectively. AI tools have shown promise in addressing some of the limitations within these frameworks. For biologics, species selection is often constrained by pharmacologic relevance and tissue cross-reactivity. AI-enabled PBPK and TCR modeling can support more informed species justification, especially when combined with transcriptomic or proteomic data or metagenomics data. However, whether these models can replace empirical evidence remains uncertain, and their acceptance depends on transparent validation and biological plausibility.

Synthetic peptides, particularly those under 40 amino acids, present a different set of challenges. These compounds often fall under small molecule guidance (ICH M3(R2)) (ICH, 2009) and may face difficulties in achieving maximum feasible dose due to solubility or formulation constraints. AI-based PBPK modeling and mechanistic de-risking can help address these issues, but questions remain about the reliability of such simulations in predicting complex *in vivo* behavior. Modified peptides, such as those that are lipidated, PEGylated, cyclized, or incorporate non-natural amino acid residues, may undergo proteolytic cleavage to form small molecule-like intermediates that engage unintended targets. These intermediates are often not characterized until unexpected *in vivo* findings emerge, highlighting a critical blind spot in traditional safety assessment. AI tools can help identify these risks early, but their predictions must be rigorously evaluated before they can inform regulatory decisions. These intermediates highlight a critical blind spot in traditional safety assessment. This is a prime use-case for AI: utilizing generative models to predict likely cleavage products and their off-target affinities before synthesis, effectively *“de-risking”* the molecule *in silico*.

Immunogenicity and organ-specific toxicities (Brennan et al., 2024) are additional areas where AI tools may offer value, though not without limitations. Furthermore, classifiers for drug-induced liver injury (DILI) (Rao et al., 2023), kidney injury (DIKI) (Rao et al., 2024), and neurotoxicity (DINI) (Behl et al., 2025), as well as tools such as NetMHCpan (Jurtz et al., 2017) and DeepImmuno (Li et al., 2021), can simulate immune activation and anti-drug antibody formation. These approaches align conceptually with the weight-of-evidence logic of ICH S8 for immunotoxicity evaluation (ICH, 2005) and offer a pathway for early hazard identification. However, their usefulness depends on the quality and relevance of the training data. For example, current models often classify compounds as DILI-positive or DILI-negative, but they do not account for dose-dependent effects, such as the threshold at which a compound becomes toxic or whether a high clinical dose might still avoid causing DILI. AI approaches that integrate dose information along with chemical and biological signatures could help fill this gap. Still, without robust validation, these tools may produce misleading results.

There is also growing interest in using AI to reduce the number of animals needed in studies. For instance, virtual control groups (VCG) created from historical data could replace live control animals in some cases (Steger-Hartmann et al., 2020; Golden et al., 2024). This approach may improve consistency and reduce costs, but it must be carefully validated to ensure it does not introduce new sources of bias or uncertainty. Interoperability across organizations and transparency in data sources are essential for building trust in these methods.

Choosing the right species for testing is another area where AI might assist drug developers. By modeling how a drug behaves in different animals, AI tools can support better decisions about which species are most relevant (Namdari et al., 2021). This could reduce unnecessary testing and improve the likelihood that results will translate to humans. However, these models must be clearly documented, biologically plausible, and easy to interpret if they are to be trusted in regulatory settings.

Overall, AI-enabled NAMs present significant opportunities, but their impact will depend on rigorous development, transparent interpretation, and alignment with regulatory requirements. While unlikely to fully replace traditional methods in the near term, these approaches can meaningfully enhance decision-making when applied appropriately and within defined contexts of use. In drug discovery and development, where adoption is shaped by regulatory conservatism and guided largely by FDA and ICH standards, greater alignment and regulatory impetus will be essential to encourage pharmaceutical companies to integrate these novel tools into submission packages. Clearer guidance is critical. As discussed in Sections 2.4 and 5, emerging frameworks like “*e-validation*” offer a pathway to bridge this gap by simulating validation studies, establishing continuous performance monitoring, and defining prospective pilot pathways for regulatory learning.

### 2.2 AI as an enabler of Green Toxicology

AI is emerging as an important enabler of Green Toxicology, particularly for rapid hazard profiling, safer-by-design prioritization, and extraction of toxicity evidence from large chemical corpora (Maertens et al., 2014; Crawford et al., 2017; Maertens and Hartung, 2018; Maertens et al., 2024c). Green Toxicology emphasizes early intervention - designing out toxicity before hazardous substances enter production or the environment - and AI can provide the scalability needed to screen large chemical spaces. Deep learning and machine learning models, including read-across-based structure/activity relationships (RASAR), can analyze millions of molecular features (Gao et al., 2025) and help prioritize compounds for cytotoxicity, genotoxicity, or endocrine-disruption concerns based on chemical structure alone.

Integrated with high-throughput screening platforms and robotics, AI facilitates automated *in vitro* testing pipelines that dramatically reduce the need for animal studies while increasing human relevance. Image recognition algorithms using convolutional neural networks (CNNs) can interpret cell morphology changes in real time, replacing manual evaluations and enabling continuous monitoring of chemical effects at the cellular level. AI also supports lifecycle-oriented chemical design by simulating the environmental and biological behavior of new compounds, guiding decision-making that accounts for both efficacy and long-term ecological safety. Furthermore, emerging initiatives such as the ONTOX project are expanding AI’s utility by developing predictive models across hundreds of endpoints and linking them to exposure-aware, non-animal safety assessment strategies (Vinken et al., 2021). Large public infrastructures such as Tox21^9^ and ICE^10^ further provide curated data, benchmarking resources, and interpretation tools that make AI-enabled Green Toxicology workflows more reproducible and comparable across organizations. Despite ongoing challenges in model interpretability, regulatory acceptance, and data quality, the synergy between AI and Green Toxicology is poised to revolutionize chemical risk assessment, enabling faster, cheaper, and more ethical safety decisions that align with sustainability goals.

### 2.3 AI as the enabler of a human exposome project

AI could help operationalize the long-envisioned Human Exposome Project (HEP), transforming it from an aspirational concept into a more data-driven program for precision public health (Hartung, 2023c). The exposome encompasses the totality of an individual’s environmental exposures - from chemical pollutants to social stressors - across the lifespan. The sheer heterogeneity and volume of these data have historically rendered comprehensive analysis difficult. AI now provides computational tools that may integrate, interpret, and act on these multidimensional data streams at scale.

Importantly, “Exposome Intelligence = EI” enables stratified risk assessments by embedding genetic, behavioral, and sociodemographic variables into predictive algorithms. This allows for individualized risk scores and tailored public health strategies-ranging from dietary and lifestyle adjustments to exposure-specific policy interventions. AI-powered hazard detection also facilitates real-time environmental monitoring via mobile sensors, alerting at-risk populations to spikes in air pollution, chemical spills, or heat stress before harm occurs. These insights depend on establishing interoperable, privacy-preserving data commons that connect public health records, biobanks, and citizen science contributions-all built on FAIR data principles to ensure reusability and cross-platform analytics. Efforts such as the NIH Data Commons and the European FAIR Environmental and Health Registry represent early steps in this direction.

To fully realize this vision, regulatory frameworks must evolve to accept AI-derived insights as valid evidence, complementing or even replacing outdated, animal-based risk assessments. Cross-sector collaborations are essential: government agencies, academia, industry, and civil society must co-develop ethical, transparent, and equitable exposome intelligence tools. Simultaneously, workforce development must prioritize data fluency, causal modeling, and algorithmic accountability to ensure that tomorrow’s environmental health leaders can translate AI outputs into policy action. This vision will require interoperable, privacy-preserving data commons that connect public health records, biomonitoring, biobanks, and citizen science contributions while adhering to FAIR data principles (Sillé et al., 2020; Sillé et al., 2024). If developed responsibly, exposome intelligence will uncover “*unknown unknowns*” in disease causation and facilitate anticipatory interventions. In doing so, AI not only enables the Human Exposome Project (Hartung, 2023a) - it redefines the frontier of public health in the 21st century.

### 2.4 Illustrative case examples and prospective pilots

Concrete examples help distinguish aspirational AI rhetoric from credible near-term use. First, virtual control groups (VCGs) are one of the most mature toxicology applications. Historical control data, when harmonized and quality-controlled, can support model-based replacement of some concurrent control animals in repeat-dose safety studies. The translational value here is pragmatic rather than futuristic: fewer animals, more consistent baselines, and faster study execution. Yet the context of use must be narrow, data provenance explicit, and prospective shadow-mode testing retained until equivalence is shown for pathology, clinical chemistry, and study interpretation endpoints (Steger-Hartmann et al., 2020; Golden et al., 2024).

Second, peptide and biologic development already offers a practical arena for AI-supported immunogenicity assessment. Sequence-based tools such as NetMHCpan and DeepImmuno can rank candidate epitopes, prioritize HLA coverage concerns, and flag design liabilities before animal or first-in-human studies. In a prospective pilot, such predictions could be locked before experimental testing and then compared with anti-drug antibody readouts, cytokine-release findings, and exposure metrics to determine whether the model is sufficiently calibrated for decision support within a defined ICH S6/S8 context (ICH, 2005, 2011; Jurtz et al., 2017; Li et al., 2021).

Third, Green Toxicology illustrates how AI can act upstream of regulation by helping developers avoid hazard rather than merely detect it later. Read-across/RASAR models can prioritize safer analogues, while generative approaches can propose candidate molecules that preserve function but reduce predicted toxicity. These ranked candidates can then be evaluated in targeted *in vitro* batteries and exposure-informed assessments, creating a prospective safe-by-design workflow with measurable success criteria such as fewer regrettable substitutions and better concordance between *in silico* ranking and follow-up testing (Maertens et al., 2021, 2024c; Luechtefeld and Hartung, 2025).

A fourth example is probabilistic risk assessment using *in vitro*-to-*in vivo* extrapolation (IVIVE) and PBPK modeling. Here, AI does not replace toxicological judgment; it helps integrate assay outputs, exposure estimates, and uncertainty into risk distributions that are more decision-relevant than binary hazard calls. A credible pilot would prespecify the target decision, benchmark the probabilistic model against established reference chemicals, and evaluate not only discrimination but also calibration and uncertainty communication (Chang et al., 2022; Maertens et al., 2022, 2025). Taken together, these examples show that the most credible route to regulatory acceptance is not a single grand replacement claim, but context-of-use-specific pilots in which AI operates alongside existing toxicological practice and earns trust prospectively.

### 2.5 Quantitative snapshot of representative AI toxicology studies

Because the present article is a perspective rather than a meta-analysis, Table 1 is not intended to be exhaustive. Instead, it quantifies a representative set of studies spanning hazard classification, mutagenicity, developmental toxicity, and immunogenicity. Dataset sizes in this sample range from about 10^3^ compounds to more than 3 × 10^4^ peptide or chemical records. In this cross-section, three of five examples reported independent external test data, whereas calibration, abstention policies, and post-deployment monitoring were far less consistently foregrounded. This pattern helps explain the central thesis of the paper: promising performance metrics are necessary, but not sufficient, for regulatory trust.

**Table 1:** Quantitative snapshot of representative AI toxicology studies.

Context/study	Dataset and evaluation design	Selected quantitative signal	Implication for regulatory use
Hazard classification Luechtefeld and Hartung (2025)	~10,000 chemicals; >866,000 properties/hazards; cross-validation across 9 hazard endpoints	Balanced accuracy 70–80% (simple RASAR) and 80–95% (data-fusion RASAR); repeat animal tests 78–96% reproducible	Large-scale hazard modeling is feasible, but applicability remains endpoint- and dataset-specific.
Ames mutagenicity Chakravarti and Alla (2019)	17,005 training compounds and 1,942 external test compounds; descriptor-free LSTM models	External BAL_ACC 0.879 and AUC 0.938 for the SMILES-based LSTM model	Strong discrimination can be achieved, but true performance depends on external chemical-space challenge sets.
Developmental screening Green et al. (2021)	1,003 ToxCast chemicals split 80:20 plus an independent external test set of 56 chemicals	Consensus model sensitivity 71.4%, specificity 95.9%, PPV 71.4%, kappa 0.673, AUROC 0.837	Useful prescreening performance, but not yet a stand-alone substitute for decision-critical testing.
Reproductive/developmental toxicity Lee et al. (2025)	4,514 compounds; stratified 5-fold cross-validation for a graph-convolution model	Test accuracy 81.19%	Promising endpoint-specific performance, but external validation is still needed for translational confidence.
Immunogenicity prediction Li et al. (2021)	>30,000 peptide–MHC observations from IEDB with 10-fold validation and independent dengue, neoantigen, and COVID datasets	Training false-positive rate 0.12 and false-negative rate 0.05; precision 0.28 in the convalescent COVID set	Large curated datasets help, but heterogeneous real-world test sets remain challenging.

## 3 The risks: bias, opacity, and validation gaps

Despite these advancements, the risks associated with uncritical adoption of AI in toxicology are substantial (Box 1) (Hartung et al., 2025a, 2025b; Bhuller et al., 2025). The most insidious among them is bias - present not only in the training data but also in how AI models are developed, validated, and interpreted. Toxicological datasets often reflect historical biases, from overrepresentation of certain chemical classes to inconsistent nomenclature and sparse metadata. If uncorrected, AI models can reinforce these patterns, giving an illusion of objectivity while concealing inherited error.

BOX 1 **Key challenges for AI use in toxicology.**

•*Data Quality and Availability* - Limited, imbalanced, or poorly curated datasets hinder reliable model training.

•*Bias and Fairness* - Historical and structural biases in training data can propagate inequities.

•*Model Interpretability* - “Black-box” algorithms lack transparency, making regulatory acceptance difficult.

•*Validation Complexity* - Traditional validation methods may not apply to dynamic, evolving AI systems.

•*Regulatory Readiness* - Frameworks and standards for AI-based decision-making are still evolving.

•*Human-AI Interaction* - Defining the appropriate level of oversight and responsibility remains unresolved.

These six challenge areas foreshadow the logic of TREAT: Trustworthiness responds mainly to data quality and bias; Reproducibility to model stability and change control; Explainability to black-box behavior; Applicability to domain fit and transferability; and Transparency to documentation, governance, and human accountability. The e-validation proposal then operationalizes these principles through iterative benchmarking, monitoring, and reporting.

Model interpretability is another major concern. Many high-performing models, particularly those based on DL, operate as “*black boxes*.” Their internal logic-how they arrive at a given prediction - remains largely inaccessible to users and regulators. This undermines one of the pillars of regulatory science: transparency. Explainable AI (xAI) is a promising field aiming to solve this problem, offering techniques like feature attribution, surrogate modeling, and saliency maps. However, these solutions are still immature and often tailored to specific model architectures, limiting their generalizability.

Traditional validation frameworks are also ill-suited to evolving AI systems. Conventional toxicological validation assumes static test methods and endpoints, while AI models continue to learn and adapt. This mismatch has regulatory implications. A validated AI model today may perform differently tomorrow as new data are incorporated. Continuous validation, or “*living validation*,” is needed-requiring robust performance monitoring systems that detect drift, retrain models where appropriate, and log changes transparently (Hartung and Kleinstreuer, 2025).

Ethical concerns also loom large. AI systems used for regulatory toxicology are increasingly intertwined with questions of data privacy, equity, and social trust. For example, if AI models are used to assess risks in vulnerable populations-such as children, pregnant individuals, or communities with high cumulative exposure-there must be confidence that these models are trained on relevant data and do not systematically under- or overestimate risk. The lack of standardized guidelines for ethical AI in toxicology remains a significant gap.

From our publication (Maertens et al., 2024b), Box 2 shows some of the challenges for the example of image-based methods.

BOX 2 **Safeguarding medical imaging in the age of AI (Maertens et al., 2024b).**

•*Data quality and provenance:* inconsistent, poorly documented

datasets

•*Bias in training data:* demographic, device, and projection-related

•*Filtered images from smartphones* distort diagnostic features

•*Pre-processing* (e.g., compression, pruning) reduces data integrity

•*Generative AI poisoning:* malicious manipulation of datasets

•*AI self-looping* (‘Model Autophagy Disorder’) degrades reliability

•*Lack of standardization* across repositories limits benchmarking

•*Mislabeled or unverified images* undermine algorithm trustworthiness

•*Scientific literature pollution* affects LLM-based medical advice

•*Vulnerability to cyberattacks* and tampering in medical image systems

## 4 A path forward: responsible AI for evidence-based toxicology

So how can we realize the benefits of AI without succumbing to the pitfalls of technological snake oil (Tsaioun et al., 2025)?

First, explainability must be built into AI models from the outset, not retrofitted after regulatory resistance. This means prioritizing model architectures that allow for traceable predictions, integrating domain knowledge through causal modeling and ontologies like AOPs, and documenting assumptions transparently. A model that performs well but cannot be explained is a scientific liability, not an asset (Hartung et al., 2025a; FDA and EMA, 2026).

Second, validation must evolve. E-validation frameworks-now under development-aim to simulate ring trials digitally, select optimal reference compounds, and assess generalizability using synthetic datasets (Hartung et al., 2024; Hartung and Kleinstreuer, 2025). Coupled with federated learning approaches, these strategies can help maintain model robustness while respecting data confidentiality.

Third, human oversight must remain central. Toxicologists should not be replaced by algorithms but empowered by them. AI should augment, not supplant, human expertise - especially in high-stakes decisions where ethical judgment, contextual understanding, and regulatory accountability are paramount (Bhuller et al., 2025; Hartung, 2024).

Finally, education is key. Toxicologists must be equipped with at least foundational understanding of AI tools, while data scientists must understand the unique demands of regulatory toxicology. Interdisciplinary training programs and cross-sector collaborations - such as those enabled by ONTOX (see text footnote 5), Tox21 (see text footnote 9), Complement-ARIE (see text footnote 8), PARC,^11^ and the Integrated Chemical Environment (ICE) (see text footnote 10) - are essential to build this capacity.

## 5 The need for validation and building trust

The promise of artificial intelligence (AI) in toxicology is immense: predictive accuracy, mechanistic insight, and human relevance at scale (Kleinstreuer and Hartung, 2024). Yet the credibility of AI-based models hinges not only on their technical performance but also on the trust they inspire (Hartung et al., 2025a). In toxicology - a field with direct implications for human and environmental health - this trust must be earned through rigorous validation and transparent governance.

### 5.1 Why validation matters

Validation serves as the scientific backbone of regulatory acceptance (Hartung, 2024). Historically, toxicological validation has relied on extensive ring trials to establish reliability and relevance. However, this traditional paradigm does not map well onto AI systems, which are dynamic, continuously updated, and often lack interpretability (Hartung and Kleinstreuer, 2025). The non-deterministic nature of many AI models-where identical inputs may yield differing outputs under evolving model states-challenges the assumptions of static validation and necessitates a shift toward frameworks that can accommodate continual performance assessment.

In toxicology, validation has traditionally emphasized four pillars: reliability, relevance, robustness, and transparency. AI complicates each of these. Its statistical logic often eludes direct mechanistic interpretation, its performance may vary with minor data or architecture shifts, and its internal decision processes can be opaque. These challenges demand a reconceptualization of what constitutes a validated method in the AI era.

### 5.2 Toward e-validation: rethinking the process

To meet this challenge, a novel framework - termed e-validation - has been proposed (Hartung et al., 2024; Hartung and Kleinstreuer, 2025). It seeks to harness AI not only as a subject of validation but also as a tool for improving validation itself. E-validation replaces rigid procedural templates with a modular, adaptive framework tailored to AI-based NAMs. It includes intelligent selection of diverse and informative reference chemicals, virtual simulations of validation studies to optimize design and detect bottlenecks, and a mechanistic validation layer that leverages AI (e.g., large language models) to identify biologically plausible links between predictions and known pathways of toxicity. E-validation should be understood as complementary to - not a replacement for - existing quality frameworks such as Good *In Vitro* Method Practice (GIVIMP) for *in vitro* methods and OECD reporting schemes for regulatory models (OECD, 2018, 2024).

Additionally, e-validation introduces AI-driven training platforms to support personalized instruction for researchers and regulators, and it culminates in centralized dashboards that visualize real-time performance metrics, automate data quality checks, and track uncertainty over time. This forward-looking infrastructure reflects the iterative nature of AI and anchors its validation in both empirical rigor and operational practicality.

A practical next step is to test e-validation prospectively in shadow-mode pilots. For VCGs, AI-derived controls can be run in parallel with conventional concurrent controls until prespecified non-inferiority criteria are met for key study conclusions. For immunogenicity models, predictions can be frozen in advance and compared with anti-drug antibody and clinical readouts across a defined HLA and dose context. For safe-by-design chemistry programs, AI-ranked substitutes can be prospectively evaluated in GIVIMP-aligned *in vitro* batteries and IVIVE/PBPK workflows. Such pilots would test not only headline accuracy but also calibration, failure modes, operational feasibility, and reviewer confidence (Golden et al., 2024; Jurtz et al., 2017; Li et al., 2021; OECD, 2018; Chang et al., 2022; Maertens et al., 2021).

### 5.3 The TREAT framework: building trust

The integration of AI into regulatory science hinges not only on performance, but on societal and institutional trust. The TREAT principle - comprising Trustworthiness, Reproducibility, Explainability, Applicability, and Transparency - offers a multidimensional approach to fostering that trust (Hartung et al., 2025a).

Trustworthiness remains the ultimate objective, though it defies simple quantification. It must be demonstrated through robust governance, sound ethical practices, and an ongoing track record of reliability. Reproducibility, while traditionally defined as yielding identical results under the same conditions, must be reinterpreted for AI as maintaining consistent predictive performance across different data sets and model iterations. Explainability is crucial for both accountability and stakeholder engagement. While high accuracy may reduce the urgency for detailed explanations in low-stakes contexts, in regulatory toxicology, understanding how predictions are derived is essential for ensuring scientific defensibility.

The applicability of AI models refers to their defined domain of use - the chemical space and biological endpoints for which predictions are valid. However, the field must strike a balance between well-defined applicability and the flexibility needed for AI systems to evolve and expand. Finally, transparency-regarding data provenance, algorithmic logic, and performance boundaries-must be maintained without unduly impeding innovation. Transparency is not only a scientific necessity but also a prerequisite for regulatory legitimacy.

Together, the TREAT framework does not prescribe fixed solutions, but rather provides a shared language and evaluative lens through which to assess the readiness of AI for regulatory deployment.

#### 5.3.1 Operationalizing TREAT across regulatory settings

The utility of TREAT increases when it is mapped to specific regulatory contexts rather than treated as an abstract checklist. Table 2 summarizes how TREAT and e-validation can be translated into evidence packages for pharmaceuticals, industrial chemicals, and public health/exposome applications, and how they interface with current guidance.

**Table 2:** Operationalizing TREAT and e-validation across different regulatory settings.

Setting	Existing regulatory anchor(s)	Illustrative AI/NAM use case	TREAT/e-validation operationalization
Pharmaceuticals/biologics	ICH M3(R2), ICH S6(R1), ICH S8, FDA/EMA good AI practice	Virtual control groups; immunogenicity prediction; PBPK/IVIVE for species selection and peptide de-risking	Lock the context of use, perform external validation, provide mechanistic justification, maintain version control, run shadow-mode pilots, and submit regulator-facing model/report cards.
Industrial chemicals/Green Toxicology	OECD QAF/QSAR principles, GIVIMP, AOP/IATA guidance	AI read-across/RASAR, safe-by-design prioritization, high-throughput screening and image analytics	Use QMRF/QPRF/QRRF-style reporting, define applicability domains, document provenance and assay quality control, report uncertainty, and monitor post-deployment performance.
Public health/exposome	Fit-for-purpose public-health decision support; FAIR and data-governance frameworks	Exposome intelligence, probabilistic risk ranking, sensor-enabled alerts	Use privacy-preserving linkage, subgroup fairness audits, calibration across populations, transparent uncertainty communication, and human oversight for interventions.

In practice, this means that TREAT does not displace existing guidance. Rather, it functions as a cross-cutting overlay: ICH guidance defines the decision context for pharmaceuticals; OECD frameworks structure model reporting, *in vitro* quality, and integrated evidence for chemicals; and newer FDA/EMA AI principles add expectations for governance and life cycle management in drug development (ICH, 2005, 2009, 2011; OECD, 2017, 2018, 2024; FDA and EMA, 2026).

#### 5.3.2 Illustrative benchmarks for TREAT criteria

To make the hypothesis more testable, Table 3 proposes illustrative, context-of-use-specific benchmarks for each TREAT element. These are not universal pass/fail thresholds; rather, they are candidate criteria that should be prospectively specified, justified, and audited for the intended regulatory purpose.

**Table 3:** Illustrative benchmarks for TREAT criteria.

Criterion	Illustrative benchmark(s)	Example evidence
Trustworthiness	Documented intended use, provenance audit, and independent bias/governance review before deployment; subgroup performance gaps prespecified.	Model card, bias audit, governance record, decision log.
Reproducibility	Stable reruns and external validation across at least two independent datasets or sites; predefined drift trigger such as a >5% drop in the primary metric.	Versioned code/data, locked model, monitoring dashboard, change-control record.
Explainability	Top drivers are biologically plausible and linked to known AOPs or mechanisms of action; local explanations remain stable for similar inputs.	Feature attribution, counterfactual examples, expert review notes.
Applicability	Explicit chemical/biological domain of use, out-of-domain flagging, and abstention rules for unsupported cases.	Applicability-domain analysis, uncertainty score, failure-mode log.
Transparency	Complete documentation of data sources, preprocessing, model updates, uncertainty, and change history.	Dataset card, model documentation, update log, submission appendix.

This emphasis on prespecification is important because an AI model can be highly accurate on average yet still fail a regulatory use case if subgroup performance, calibration, documentation, or out-of-domain behavior is inadequate.

#### 5.3.3 Minimum dataset and model-evaluation dossier

**Figure 3:**
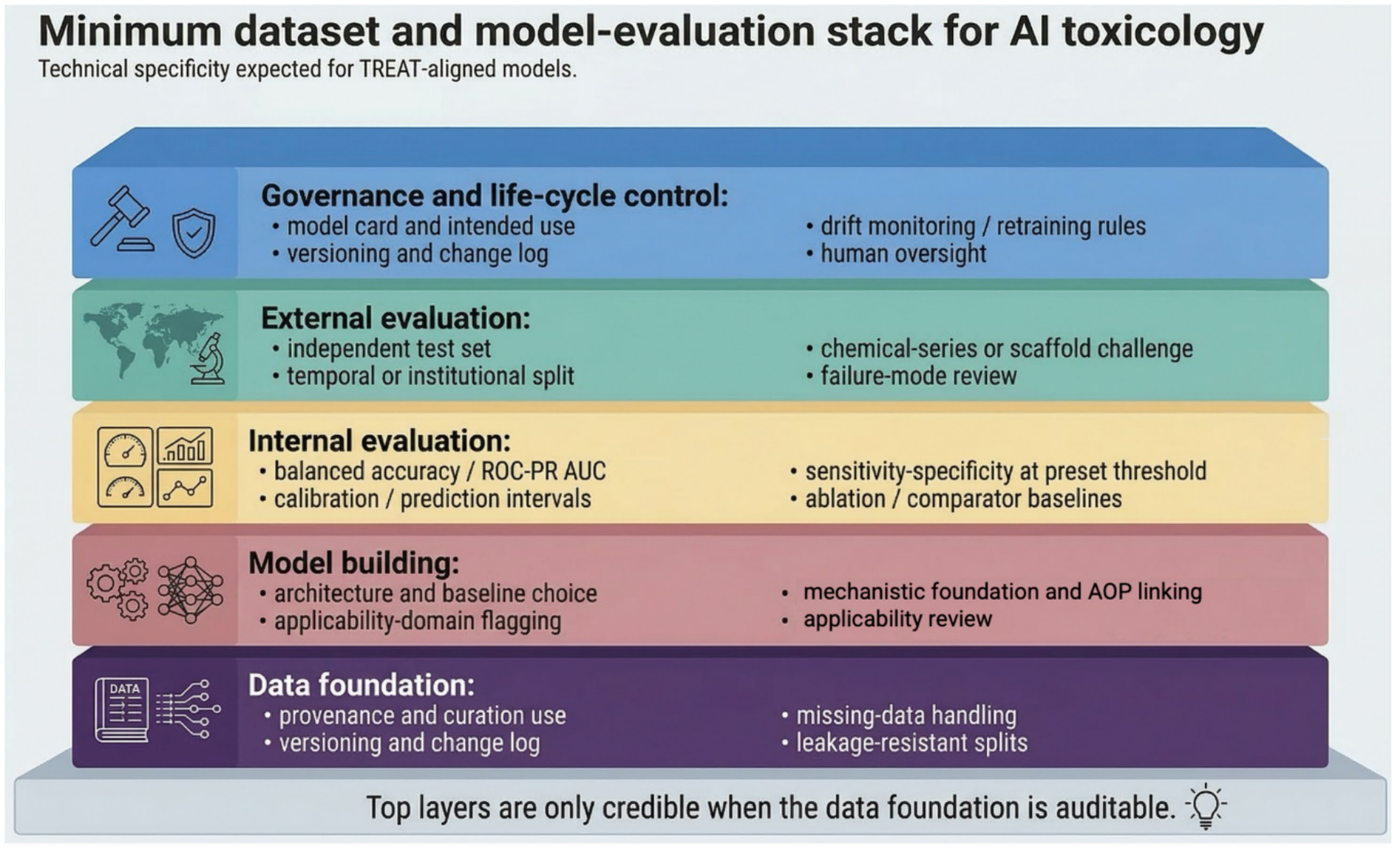
Minimum dataset and model-evaluation stack for AI toxicology models. The schematic highlights the technical elements that should be documented for TREAT-aligned models, from provenance and split strategy to external validation, calibration, applicability domain, and governance. Figure draft created with Gemini Nano Banana 2.

Seen this way, TREAT becomes operational only when it is attached to a minimum technical dossier (Figure 3). At a minimum, a regulatory-facing AI toxicology model should disclose dataset provenance and curation rules, chemistry- or study-aware train/validation/test splitting, missing-data handling, class balance, comparator baselines, and an explicit context of use. Random record-wise splits alone are often insufficient, because closely related chemicals or assay conditions can appear in both training and test sets and inflate apparent performance.

For classification models, discrimination metrics (e.g., balanced accuracy, ROC-AUC or PR-AUC) should be accompanied by sensitivity, specificity, PPV/NPV, calibration at prespecified decision thresholds, and uncertainty estimates. For continuous endpoints, MAE/RMSE should be paired with prediction intervals. External validation should, where possible, be temporal, institutional, or chemical-series based, and the dossier should include applicability-domain analysis, failure-mode review, and a versioned change log. These elements are not administrative overhead; they are the technical substrate of TREAT.

### 5.4 The role of continuous validation and companion agents

Because AI models evolve over time, static validation is no longer sufficient. Instead, continuous validation mechanisms must be implemented (Hartung and Kleinstreuer, 2025). A particularly promising innovation is the use of companion AI agents that monitor performance post-deployment. These agents autonomously ingest new data, assess model quality, initiate retraining when appropriate, and flag changes in prediction outcomes. In doing so, they transform validation into a living process-one that adjusts to emerging knowledge and data while maintaining consistency with prior evaluations.

These agents are envisioned to operate through centralized dashboards that provide transparent access to current model versions, performance metrics, applicability domains, and uncertainty estimates. In addition, they could alert users to relevant changes, enabling both retrospective reevaluation and forward-looking oversight. This infrastructure not only enhances technical performance but also reinforces trust through auditability and user engagement.

### 5.5 Human-AI collaboration and the importance of oversight

AI must not replace human toxicologists but augment them. Human oversight remains indispensable, particularly in contexts involving ethical judgment, contextual knowledge, or regulatory consequences. Toxicologists must be trained to understand AI outputs, question their limitations, and integrate them with domain-specific knowledge.

Conversely, data scientists must be educated in the unique regulatory and ethical challenges of toxicology. Interdisciplinary education-spanning machine learning, risk assessment, regulatory science, and ethics-should be prioritized to ensure that human-AI collaboration is both scientifically sound and socially responsible.

### 5.6 The ethics of trust

Trust is not only a matter of technical performance or procedural compliance; it also implicates fairness, equity, and inclusion (Bhuller et al., 2025; Hartung, 2024). AI systems trained on historical toxicity data may inherit and perpetuate biases-whether demographic, methodological, or chemical class–specific. Without careful bias auditing, models may under predict risks for underrepresented populations or chemical categories.

To build equitable systems, datasets must be curated to reflect the full diversity of exposures, populations, and contexts. Privacy must also be safeguarded, especially as AI begins to integrate personal exposure data, social determinants, and genomics into risk models. Ethical frameworks must evolve in tandem with technical ones, ensuring that trust in AI extends beyond the lab and into the public sphere.

Trust in AI-enabled toxicology must be cultivated-not presumed. This requires a reconceptualization of validation to reflect the dynamic, non-linear, and probabilistic nature of AI models. E-validation offers a roadmap for aligning methodological rigor with operational flexibility. The TREAT framework provides ethical and epistemic anchors for trust. And companion validation agents may enable regulatory systems to keep pace with technological innovation. In the end, validation is not merely a gateway to adoption-it is the process through which trust is built, sustained, and justified.

### 5.7 Boundary conditions and limitations

Our hypothesis is not endpoint-agnostic. AI applications are most likely to achieve early regulatory traction when the context of use is narrow, the endpoint is relatively well characterized, training data are curated and representative, and the model can be locked and externally validated. Acceptance will likely be slower for rare events, long-latency outcomes, poorly annotated biologic liabilities, and continuously self-updating foundation models that lack version control or auditable provenance. Likewise, models trained primarily on limited chemical spaces or single-platform data should not be extrapolated to novel modalities, mixed exposures, or underrepresented populations without new evidence. These boundary conditions clarify that TREAT and e-validation do not eliminate uncertainty; rather, they provide a disciplined framework for deciding when AI evidence is strong enough for a defined regulatory use and when it should remain supportive rather than decisive (Hartung and Kleinstreuer, 2025; OECD, 2024; FDA and EMA, 2026).

## 6 Conclusion: no magic, just better science

AI is unlikely to replace toxicologists, but toxicology practice is likely to change substantially as AI becomes embedded in data curation, prediction, and evidence synthesis. The same is true for regulators, risk assessors, and safety scientists: the relevant challenge is not whether AI exists, but whether the field can use it critically and responsibly.

The snake oil metaphor therefore remains useful only as a caution against unsupported claims. AI is not magic. It is a set of methods whose value depends on data quality, validation, and context of use. Used judiciously, it can help propel toxicology toward a more predictive, transparent, and human-relevant future; used uncritically, it can create synthetic certainty where uncertainty should instead be acknowledged.

Beyond technical frameworks, responsible AI in toxicology also requires governance mechanisms that match its societal impact (Box 3). As outlined in recent policy commentary, AI - like the data on which it depends - should be treated as a strategic societal asset, subject to transparent oversight and stewardship rather than left “*in the wild*” (Hartung, 2023a). A global, use-case-based regulatory approach grounded in FAIR data principles could complement the TREAT framework (Hartung et al., 2025a), ensuring that transparency and applicability are not just aspirational but enforced. Such measures, including clear disclosure of AI involvement in decision-making, would align toxicology with emerging international AI governance norms and strengthen public trust. This policy perspective situates e-validation and TREAT within a broader ecosystem of accountability, where scientific rigor is reinforced by proactive regulation.

BOX 3 **Policy perspective - governing AI in toxicology.**

•*AI as a strategic societal asset* – Treat AI and its underlying data as resources requiring stewardship, not unchecked exploitation.

•*Global, use-case–based regulation* – Develop governance tailored to specific applications, mirroring proposals for an *International Data and AI Agency*.

•*FAIR data principles* – Ensure data are Findable, Accessible, Interoperable, and Reusable, reinforcing the *Applicability* and *Transparency* pillars of TREAT.

•*Mandatory AI disclosure* – Require explicit indication when AI systems are used in decision-making, aligning with transparency norms.

•*Integration with scientific frameworks* – Couple regulatory oversight with technical safeguards like *e-validation* and continuous performance monitoring to ensure both rigor and accountability.

## 7 Beyond the snake-oil analogy

AI in toxicology is not best understood as modern snake oil. It is better viewed as a powerful but double-edged enabling technology: valuable when it converts heterogeneous data into reproducible evidence, risky when it produces unjustified certainty.

Data are often described as the new oil, and AI can indeed transform those data into predictive and mechanistic insight. But that analogy is incomplete. Without rigorous curation, external validation, and human oversight, AI can also amplify bias, obscure uncertainty, and create an illusion of precision.

The relevant scientific question is therefore not whether AI is inherently transformative, but under which conditions it becomes trustworthy enough for a defined regulatory or research context.

In that sense, AI is neither panacea nor fraud. Its value will depend on whether toxicology embraces it as an evidence-generating technology bounded by TREAT, e-validation, and fit-for-purpose human oversight.

AI should thus be judged neither by hype nor by fear, but by evidence.

When evaluated in that way, AI becomes a demanding but genuinely useful addition to the toxicological toolbox.

Whether AI merely accelerates existing workflows or helps establish more predictive and ethical toxicology will depend on the quality of the data, the rigor of the validation, and the discipline with which the field defines its contexts of use.

## Data Availability

The original contributions presented in the study are included in the article/supplementary material, further inquiries can be directed to the corresponding author.

## Footnotes

1 https://dictionary.cambridge.org/dictionary/english/snake-oil and https://www.britannica.com/dictionary/snake-oil

2
https://www.ihi.europa.eu/projects-results/project-factsheets/vict3r

3
https://www.ebtox.org

4
https://www.sysrev.com

5
https://ontox-project.eu

6
https://www.risk-hunt3r.eu

7
https://panoramix-h2020.eu

8
https://commonfund.nih.gov/complementarie

9
https://tox21.gov/overview/about-tox21/

10
https://ntp.niehs.nih.gov/whatwestudy/niceatm/comptox/ct-ice/ice

11
https://www.eu-parc.eu/

## Glossary

NAMs: New approach methodologies used to generate human-relevant, non-animal evidence for hazard and risk assessment
Read-across/RASAR: Prediction of a target chemical from similar source chemicals, often combining structural and biological similarity in machine-learning workflows; RASAR = read-across-based structure activity relationship
AOP: Adverse outcome pathway; a mechanistic framework linking a molecular initiating event to downstream key events and an adverse outcome
IVIVE: In vitro-to-in vivo extrapolation; translation of *in vitro* bioactivity or kinetics into in vivo dose metrics
PBPK: Physiology-based pharmacokinetic modeling; mechanistic simulation of absorption, distribution, metabolism, and excretion across tissues
ProbRA: Probabilistic risk assessment; risk characterization expressed as distributions and uncertainty rather than binary hazard calls
e-validation: A proposed digitally enabled validation framework using simulation, adaptive benchmarking, and continuous performance monitoring for AI-based NAMs
TREAT: Trustworthiness, Reproducibility, Explainability, Applicability, and Transparency; a framework for judging whether AI evidence is fit for regulatory use
